# Role of the Zeta Method in Intracytoplasmic Sperm Injection Outcomes in High Sperm DNA Fragmentation in Oligoasthenozoospermic Men

**DOI:** 10.22086/gmj.v0i0.1107

**Published:** 2018-06-12

**Authors:** Maryam Sefidgar Tehrani, Malihe Amirian, Mohsen Jalali, Armin Attaranzadeh, Alireza Fazel, Alireza Ebrahimzadeh-bideskan

**Affiliations:** ^1^Department of Anatomy and Cell Biology, School of Medicine, Mashhad University of Medical Sciences, Mashhad, Iran; ^2^Department of Reproductive Medicine and Gynecology, Milad Infertility Center, Mashhad, Iran; ^3^Department of Reproductive Medicine and Gynecology, Armaghan Infertility Center, Mashhad, Iran; ^4^Microanatomy Research Center, School of Medicine, Mashhad University of Medical Sciences, Mashhad, Iran

**Keywords:** Sperm Injection, Intracytoplasmic, DNA Damage, Male Infertility

## Abstract

**Background:**

New methods are needed to optimize intracytoplasmic sperm injection (ICSI) outcomes in oligoasthenozoospermic (OAS) men. We evaluated the level of DNA fragment index (DFI) in OAS men and its impact on ICSI outcomes. In addition, we used the zeta potential method for sperm selection to investigate the efficacy of this technique in improving ICSI outcomes.

**Materials and Methods:**

This cross-sectional study was performed on 95 couples. Sperm parameters and sperm DNA fragmentation (SDF) were measured. The couples were divided into the following 3 groups: group I (n=30) where SDF was between 15% and 30%, and routine sperm was selected on the basis of motility and morphology; group II (n=34) where SDF was more than 30%, and the routine sperm selection method was applied on the basis of motility and morphology; and group III (n=31) where SDF was more than 30%, and the sperm selection was performed on the basis of the zeta method. The fertilization rate, embryo development, embryo quality, and implantation rate were evaluated in these 3 groups.

**Results:**

The fertilization rate was significantly higher in group I compared with group II (P<0.05). The embryo development rate in group I was significantly higher than that in group II (P<0.001) and group III (P<0.05), and it was significantly lower in group II compared with group III (P<0.05). The embryo quality was higher in group III compared with group II (P<0.01). The implantation rate in group I was significantly higher than that in group II (P<0.05) and group III (P<0.05).

**Conclusions:**

The present study indicated that a higher level of SDF has an adverse effect on the ICSI outcome. Furthermore, the zeta potential technique can be a useful method for sperm selection in OAS men.

## Introduction

Infertility is a well-known universal problem, and, in the recent years, its prevalence has increased throughout the world. According to the World Health Organization (WHO) reports, the mean prevalence of infertility around the world is about 9% to 12%, and the infertility rate of men is about 50% of all infertility cases [1]. Deficiencies of semen and semen quality could be regarded as the main contributing factors of male infertility [2].

Male fertility is evaluated with the help of semen analysis, which includes sperm count, motility, and morphology. However, it yields limited information with regard to male infertility [3]. Some research results have shown that infertile men with normal sperm parameters have sperm DNA fragmentation (SDF), which affects the male fertility potential. Also, several studies have indicated that there is a higher amount of sperm DNA fragmented in male infertility [4-6]. Intracytoplasmic sperm injection (ICSI) is one of the assisted reproductive technologies (ARTs) that is used for the treatment of male infertility. According to the International Committee for Monitoring Assisted Reproductive Technologies (ICMART) reports, 63% of all ART cycles use ICSI, which is on the rise today. However, the safety measures of this technique have remained unclear [7]. Several reports indicate that a higher extent of SDF leads to the insemination of undesirable sperms with DNA. This causes poor prognosis and failed ICSI cycles in men, resulting in a higher risk of congenital disabilities in their offspring [8].

Therefore, use of the zeta method should be selective, and different aspects of using this method with regard to male reproductive dysfunction should be studied. Sperm selection may play a critical role in the use of ICSI technique. So in ICSI, the physiological barriers of sperm selection for fertilization are discarded. However, in the conventional fertilization method, due to the role of motility and morphology of sperm in fertilization, sperms are normally selected. In the ICSI technique, sperm is injected into the cytoplasm of mature oocyte without having any prior knowledge about sperm DNA integrity that impacts on male fertility potential [9]. Despite the disagreement over the routine use of the SDF test in evaluating the infertility of men, the American Society for Reproductive Medicine has confirmed that performing the SDF test can provide beneficial clinical information for intrauterine insemination, in vitro fertilization (IVF), and ICSI [9, 10].

Hence, the sperm chromatin integrity assay test must be conducted, especially in OAS men, which provides reliable information regarding the DNA and chromatin integrity of sperm. New approaches have emerged with the provision of more effective information in sperm DNA integrity to evaluate the amount of SDF, for example, the sperm chromatin dispersion (SCD) test [11, 12].

However, it should be noted that the SCD test should not be used in treating a couple. This test has a diagnostic aspect that allows us to know the causes of male infertility only. In clinical applications, it is important to select sperm with high chromatin integrity for insemination with oocyte [13].

Sperm with DNA fragmentation can cause miscarriage and recurrent pregnancy loss, and sperm selection based on the motility and morphology may lead to the insemination of undesirable sperm with DNA damage, which can cause poor prognosis and failed ICSI cycles [14].

Based on the performance and identification of the healthy membrane of mature sperm, several methods of sperm selection have been suggested. One of the procedures used for sperm selection is the zeta method that is based on surface charge. It is a simple method for selecting mature sperm that uses the negative charge property of -16 to -20 mV due to the sialic acids that are naturally present on the mature sperm membrane [15]. Sperm selected on the basis of the zeta potential of membrane surfaces indicates high chromatin integrity [16, 17].

Here we studied the relation between the amount of SDF and the fertilization rate, embryo development (ED), embryo quality, and the success rate of implantation in the OAS men after ICSI. Also, we compared the ICSI outcomes between the routine sperm selection method and the zeta method in a high SDF group.

## Materials and Methods

This cross-sectional study was conducted on 95 couples having their first or second male infertility factor and who were referred to the Milad and Armaghan Infertility Centers in Mashhad, Iran, from April 2016 to February 2017. All patients filled in the informed consent form. The Ethics Committee of Mashhad University of Medical Sciences (Code No. IR.mums.fm.REC.1394.320) approved the present study.

### Inclusion and Exclusion Criteria

The inclusion criteria were as follows: (1) infertile (or OAS) men receiving ICSI for the first or second time and having a history of the duration of infertility of 2 to 10 years; (2) the sperm count of the males should be between 5×106 and 14×106/mL in natural ejaculation; (3) the sperm motility rate should be below 32%, and the normal morphology rate should not be more than 30%; (4) the age of infertile men should be below 40 years; and (5) females should be aged between 20 and 37 years, have no infertility problems, and have 6 to 7 mature oocytes. The exclusion criteria were as follows: (1) females younger than 19 or older than 38 years with any ovarian problems or endometriosis and (2) males with any abnormality such as leukocytospermia.

### Analysis of Semen

After 3 to 4 days of sexual abstinence, samples of semen were collected through masturbation. The analysis of semen was carried out based on the WHO criteria [18]. Then each semen sample with the listed criteria was divided into 2 aliquots for DNA fragmentation measurement and sperm preparation by the density gradient centrifugation (DGC) method for ICSI. According to the amount of SDF and sperm selection techniques, the couples were divided into 3 groups as follows: group I (n=30) where SDF was between 15% and 30%, and sperm selection was done on the basis of motility and morphology (routine); group II (n=34) where SDF was more than 30%, and sperm selection was done on the basis of motility and morphology (routine); and group III (n=31) where SDF was more than 30%, and sperm selection was done using the zeta method for ICSI.

### Preparation of Semen by Density Gradient Centrifugation

In a 14-mL tube, the 80/40 gradient (sperm washing medium, PureCeption, SAGE, USA) was prepared by adding 2 mL semen and centrifuging it for 20 min at 200 g. Because the pellet should be undisturbed, the supernatant was removed. The pellet was washed for 5 min (at 200 g) in a warmed medium and mixed with 1 mL of sperm. The last coated pellet was mixed with a sperm of 0.5 mL, and the supernatant was removed. Thereafter, incubation was done with the help of a carbon dioxide incubator at 37°C for 30 min. The upper layer was separated and used for ICSI [19].

### Sperm Chromatin Dispersion Test

**Figure 1:**
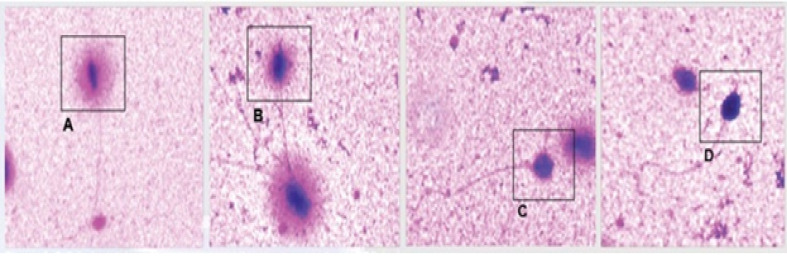
Sperm chromatin dispersion test: **(A)** nucleus with a large-sized halo, **(B)** nucleus with a medium-sized halo, **(C)** nucleus with a small-sized halo, and **(D)** nucleus without a halo. The nuclei with large- to medium-sized halos denoted a sperm with nonfragmented DNA, and the nuclei with small-sized and no halos represented a sperm with fragmented DNA (magnification = 100×, Diff–Quik staining).

The DNA fragmentation index (DFI) was measured by using the SCD test. For this, the sperm DNA Fragmentation Assay Kit (Avicenna) was used, and the manufacturer’s protocol was followed. Briefly, after the incubation of semen samples, semen smear was prepared, and then the slides were immersed in the Diff-Quik stain solution and observed with the help of a bright-field microscope. In total, 300 sperms were evaluated on each slide manually, in terms of the halo size and dispersion pattern. This evaluation was done according to the Fernandez *et al*. method. In this method, the sperm with large- and medium-sized halos of nuclei indicated nonfragmented DNA, and small-sized halos of nuclei and nuclei without halos indicated fragmented DNA [12] (Figure-1).

### Sperm Selection by the Zeta Method

The zeta method was performed on the basis of the Chan *et al*. method. In short, samples of semen were diluted to reach the concentration of 5 mL of sperm per mL in the tubes. Thereafter, the prepared solutions were centrifuged, and the supernatants were removed. Then 1 mL serum in a free medium was mixed with the remaining pellets in the tubes. After that, the tubes were placed inside a latex glove up to their caps. While holding their caps, the tubes were rotated quickly for 2 or 3 times and then pulled out rapidly. We kept tubes still for 1 min so that the sperm was stuck to them. Thereafter, these tubes were centrifuged for 5 min at 200 g. Then the medium and pellet were discarded for the separation of the nonadhering sperm and other cells. To neutralize the charge of the tube wall to detach the adhering sperm, the tube surfaces were washed with 0.2 mL of Ham’s F10 + FCS 10%. As a result of this, the adhering sperm was detached and collected at the bottom of the tubes to use for the insemination of the oocyte [15].

### Ovarian Stimulation

Ovarian stimulation was performed, and oocytes of 18-mm diameter were collected with the help of a transvaginal ultrasound-guided needle [14]. At least 6 to 7 oocytes were obtained from each woman. The collected oocytes were in the same cell meiotic division, were at the metaphase II stage, and prepared for ICSI.

### Intracytoplasmic Sperm Injection

The retrieved oocytes were incubated in the Flushing Medium (Origio). Then, they were denuded with a brief exposure to Hyadase (Origio). The simultaneous semen preparation and immobilization method was performed in polyvinylpyrrolidone (PVP) (Origio) [20]. Thereafter, the sperm was injected into the oocyte with the help of an inverted microscope equipped with micro-injectors.

### Assessment of Fertilization as well as Embryo Quality and Development

Fertilization was assessed 16 to 20 hours after insemination and confirmed by the presence of 2 pronuclei. The embryo quality was evaluated using an embryo quality scoring system of 1 to 4, which was based on the symmetry of blastomer size, quality of cytoplasm, and presence of cytoplasmic fragment. The ED rating was evaluated using the method described by Cummins *et al*. after 3 days of insemination [21]. After the third day of fertilization, 3 good-quality embryos were transferred to the mother’s uterine. The implantation was confirmed by the level of beta-human chorionic gonadotropin (β-hCG), with more than 10 IU, and by the observation of the gestational sac via ultrasound imaging 4 to 5 weeks after embryo transfer [20].

### Statistical Analysis

The Kolmogorov–Smirnov test was used to examine the normality of variables in the 3 groups. Then, one-way analysis of variance and Tukey’s statistical tests were performed for comparing parametric variables with the normal distribution. In the remaining cases, the equal nonparametric Kruskal–Wallis, and Mann–Whitney tests were performed. The statistical significance level was set at P<0.05.

## Results

In this study, all 95 men participants were infertile (OAS men). The semen parameters, including the concentration, motility, and morphology of sperm, as well as the age of patients, are shown in Table-1. According to sperm DFI results, in 30 cases (n=30), sperm DFI was between 15% and 30% (group I). In 65 cases, sperm DFI was more than 30%. According to the method of sperm selection for insemination, these cases (DFI > 30%) were divided into 2 groups: in one group (group II), sperm selection was performed using the routine method, whereas, in another group (group III), it was performed using the zeta method.

**Table-1:** Semen Analysis and Couple’s Clinical Characteristics in Group I, Group II, and Group III

Parameter Mean (SD)	Group I Mean (SD)	Group II Mean (SD)	Group III
Sperm concentration × 10^6^ 12.51 (1.64)	10-14 11.06 (1.98)	5-14 11.33 (2.23)	7-14
Sperm motility 12.10 (3.46)	5-17 14.50 (2.92)	10-20 11.33 (3.58)	5-18
Normal morphology (%) 36.00 (3.08)	32-42 38.80 (3.92)	35-45 38.80 (3.92)	32-45
Female age (years) 30.14 (3.01)	25-35 31.65 (2.25)	28-35 30.53 (3.50)	27-37
Male age (years) 35.37 (3.07)	30-40 33.20 (3.12)	28-38 31.00 (4.08)	25-39

The evaluation and comparison of the fertility rate, ED rate (EDR), embryo quality, and implantation rate in all the 3 groups are shown in Table-2. The fertilization rate in group I was significantly higher compared with group II (P<0.05), whereas no significant difference was observed between group I and group III, and between group II and group III (Table-2). The results indicated that we can compensate the high level of SDF in ICSI using the zeta method with DGC, resulting in group III.

**Table 2:** Comparison of Fertilization Rate, Embryo Quality, Embryonic Development, and Implantation Rates Between Groups

**Parameters**	**Group I**	**Group II**	**Group III**	**P-Value**		
	**Mean (SD)**	**Mean (SD)**	**Mean (SD)**	**I vs. II**	**I vs. II**	**II vs. III**
**Fertilization rate**	0.89 (0.104)	0.80 (0.117)	0.83 (0.137)	0.004*	0.079	0.369
**ED** ^a^ ** of 4-cell on day 3**	0.21 (0.084)	0.40 (0.090)	0.31 (0.137)	0.000***	0.005*	0.022
**ED** ^b^ ** of 8-cell on day 3**	0.82 (0.15)	0.61 (0.127)	0.72 (0.236)	0.000***	0.028*	0.047*
**EQ of 4-cell (grade IV)**	0.90 (0.20)	0.60 (0.205)	0.73 (0.253)	0.000***	0.005*	0.003*
**EQ of 4-cell (grade III)**	0.10 (0.20)	30.43 (0.21)	0.27 (0.253)	0.004*	0.005*	0.036*
**EQ of 8-cell (grade IV)**	0.80 (0.129)	0.67 (0.139)	0.73 (0.168)	0.000***	0.082	0.012*
**EQ of 8-cell (grade III)**	0.19 (0.123)	0.34 (0.129)	0.28 (0.168)	0.031*	0.000***	0.155
**Implantation rate**	0.47 (0.507)	0.24 (0.431)	0.42 (0.508)	.048*	0.702	0.020*

The EDR was evaluated on the basis of the number of fertilized eggs (zygote) on the third day after fertilization. Our results showed that there were 2 ED stages, including 4- and 8-cell embryos on the third day after fertilization. In 8-cell embryos, the development rate and growth of embryos were fine. However, in 4-cell embryos, there was a delay in growth and development. EDR in 4-cell embryos was compared for the 3 groups. In comparison, it was found that EDR in group II was significantly higher than that in group I (P<0.001) and group III (P<0.01), but there was no significant difference between EDR in group II versus group III. The results of EDR in 8-cell embryos showed that it was higher in group I in comparison with that in group II (P<0.001) and group III (P<0.05). Also, EDR was significantly lower in group II in comparison with that in group III (P<0.05) (Table-2). It could be seen from the table that despite the high level of SDF, EDR in group III improved in comparison with group II.

The results showed 2 grades of embryo quality—grade IV and grade III—in 4- and 8-cell embryos. The embryo quality in 4-cell embryos showed that grade IV was significantly higher in group I in comparison with group II (P<0.001) and group III (P<0.01). Also, the results showed that grade IV was significantly higher in group III in comparison with group II (P<0.01).

The embryo quality in 4-cell embryos showed that grade III was significantly higher in group II in comparison with group I (P<0.001) and group III (P<0.01). The results also showed that grade III was significantly higher in group II in comparison with group II (P<0.05). The obtained results of embryo quality assessment in 8-cell embryos showed that grade IV was significantly higher in group I in comparison with group II (P<0.001) and group III (P<0.01). However, in terms of grade IV, no significant difference was observed between group I and group III.

The results showed that, despite the high level of SDF, the embryo quality improved in group III compared with that in group II.

Also, the results of embryo quality in 8-cell embryos showed that grade III was significantly higher in group II compared with group I (P<0.001) and in group III in comparison with group I (P<0.01). However, in terms of grade III, no significant difference was observed between group II and group III (Table-2).

Our results showed that the implantation rate was significantly higher in group I compared with group II (P<0.05) and in group III compared with group II (P<0.05). However, no significant difference was observed between group I and group III (Table-2). The results revealed that the outcomes improved significantly when zeta method was used for sperm selection in group III.

## Discussion

Sperm chromatin condensation is a process characterized by sensitivity and complexity. Sperm chromatin packaging takes place during spermiogenesis. In this process, at first, protamines replace histones in a chromatin structure, leading to a change in the head of sperm [22]. Nuclear condensation results in sperm resistance to DNA damage, which may cause sperm passing through the female genital tract; therefore, it plays a protective role. Furthermore, due to the hydrodynamic force, the head of sperm can better move and penetrate through the zona pellucida of the ovum. In this regard, the component of chromatin and DNA strands supercoiled around the protamines of sperm DNA is related to fertility potential [22].

SDF is probably caused by a defect in sperm chromatin packaging or abnormalities in the natural process of apoptosis [13, 14]. According to Chohan *et al*. [23], the most common causative factors for SDF are genetics, oxidative stress, smoking, and exposure to environmental or industrial toxins. Therefore, SDF is an important factor for infertility of males, pregnancy outcomes, and high risk of miscarriage.

According to the literature, DNA fragmentation can occur in infertile males with morphologically normal spermatozoa [24]. Several studies have indicated a high level of DNA fragmentation in men who were infertile compared with the fertile ones [25].

We concluded that the fragmentation of DNA was significantly high in males’ infertility in cases of OAS. Likewise, in a study that was conducted by Oehninger *et al*. in men with OAS, the level of SDF was reported to be dramatically more in men with OAS in comparison with their healthy counterparts [26].

One of the procedures that is used routinely in the ART lab and leads to the selection of sperm with low DFI is the DGC [27]. We used this method for all cases because the levels of DNA fragmentation in 68% of the cases were more than 30%.

The results indicated that ICSI outcomes were significantly better in group III than that in group II, indicating that the DGC procedure alone cannot improve the ICSI outcomes in a high level of SDF cases, and there’s a need to use another sperm selection method accompanied by DGC.

Naturally, successful fertilization occurs when a sperm crosses the natural (physiological) obstacles around the oocyte. These obstacles are bypassed during the ICSI, which is used to treat male infertility. In our study, despite the high level of SDF, the fertilization rate was high, whereas in a research carried out by Breznik *et al*., a negative relationship was noted between the results of the halo test and the fertilization rate after IVF [3].

This discrepancy between our results and that of the above-mentioned study might be due to the fact that in the ICSI technique, the sperm is injected into the oocyte, and the fertilization evolution and embryogenesis occur regardless of the chromosomal defects, low sperm motility, poor sperm-zona pellucida binding, and incomplete acrosome reaction [28]. In other words, infertility caused by the above-mentioned factors can be managed by the employment of ICSI because the natural process of fertilization is eliminated and replaced in this method.

Moreover, in line with some of the previous studies, Seli *et al*. [29] proposed that the fertilization ability of the sperm not only depends on the sperm DNA damage rate but also is related to the ability of oocytes to ameliorate the sperm DNA damage. It could be concluded that the high level of fertilization rate depends on the amount of DNA damage that can be repaired by oocyte. Furthermore, it is suggested that the paternal genome can be activated after the 4-cell stage, and until then, ED is controlled by mRNA inherited from the maternal genome [30], which might justify the contradictory results of the previous studies in this regard.

Studies have shown that the selected sperm based on zeta capacity indicates a low level of DNA damage [16, 17, 31]. Esfahani *et al*. [17] showed that the DGC/zeta method ameliorates the percentage of good-quality embryos and pregnancy rates compared with the DGC technique. Also, they reported that in the offspring, after using the zeta method of sperm selection, the female sex was significantly higher. Also, according to the report of Duarte *et al*., in the zeta potential technique of sperm selection, the sperm was selected with low DFI for ICSI, leading to improved EDR [30].

Our analysis of ED and embryo quality was in line with the previous studies in this field, and, despite the high level of DNA fragmentation in group III that used the DGC/zeta, we observed a higher degree of ED and embryo quality.

According to the results of this study, the implantation rate was higher in group I compared with group II. These findings support the hypothesis of a negative relationship between SDF and the implantation rate after ICSI. In addition, the results of Zini *et al*. study revealed that oocyte fusion with damaged sperm DNA might lead to a high risk of miscarriage [32], and in research conducted by Wang *et al*, it was declared that if the patients had a high sperm DFI, then the loss rate of pregnancy was higher (defined as spontaneous miscarriage/biochemical pregnancy) [33]. However, in patients of group III where the zeta method was used for sperm selection, despite a high level of sperm DFI, the implantation rate improved. These results may be compared with other research studies in this field [17, 31].

## Conclusion

It was concluded that the DNA fragmentation has an important role in clinical outcomes of ICSI, and the zeta method of sperm selection may be a helpful procedure to acquire better clinical outcomes of ICSI in OAS men.

## Conflict of Interest

None declared.
